# Financial Decision‐Making, Financial Literacy, and Cognitive Function: Evidence From the PATH Through Life Study

**DOI:** 10.1002/gps.70248

**Published:** 2026-08-17

**Authors:** Htet Lin Htun, Craig Sinclair, Ranmalee Eramudugolla, Kaarin J. Anstey

**Affiliations:** ^1^ School of Psychology University of New South Wales Sydney New South Wales Australia; ^2^ Neuroscience Research Australia (NeuRA) Sydney New South Wales Australia; ^3^ UNSW Ageing Futures Institute University of New South Wales Sydney New South Wales Australia

**Keywords:** cognitive ageing, education, episodic memory, executive function, financial decision‐making, financial literacy, older adults

## Abstract

**Objectives:**

(1) To describe financial decision‐making experienes and financial literacy, and (2) to examine associations between multiple cognitive functions and financial literacy, including whether these associations are modified by gender and education.

**Methods:**

We analysed cross‐sectional data from Wave 5 of the PATH Through Life Study, including 1036–1440 adults aged 60–66 years (mean age 62.5 years [SD 1.5]; 53% women). Subjective financial literacy was assessed using self‐rated knowledge, and objective financial literacy using a PRIDIT‐weighted score derived from four items. Associations between ten cognitive measures and financial literacy were examined using multivariable linear regression, adjusting for sociodemographic characteristics. Interactions with gender and education were assessed.

**Results:**

Overall, 69% of participants had made a major financial decision, and 22% had acted as substitute decision‐makers during the previous 5–6 years. Self‐rated financial literacy was high (mean 5.0 [SD 1.2] on a 7‐point scale). Objective financial literacy was lower, especially for risk diversification (56% correct) and bond pricing (27% correct). Subjective financial literacy was associated only with self‐reported memory impairment. Objective financial literacy was associated with all cognitive function tests except self‐reported memory impairment. Associations between certain cognitive functions and objective financial literacy were weaker among participants with higher education. No gender‐based interactions were observed.

**Conclusions:**

Better cognitive function was associated with higher objective financial literacy, whereas subjective financial literacy showed no association with cognitive performance. These findings highlight the importance of cognitive function for financial literacy in later midlife to early older age adults.

## Key Points

- Financial decision‐making was common in this cohort: 7 in 10 made major decisions, and 1 in 5 acted as substitute decision‐makers.
- Objective financial literacy was associated with multiple cognitive functions, whereas subjective literacy was associated only with self‐reported memory impairment.
- Associations between cognitive performance and objective financial literacy were weaker among participants with higher educational attainment.

## 1 Introduction

Adverse financial experience among older adults is a growing global concern. A meta‐analysis of 52 studies from 24 countries estimated that 6.8% of older adults experience financial exploitation [1]. This corresponds to ∼75 million (1 in 15) people aged 60+ years each year, with substantial economic losses reported annually. In the United States alone, annual losses from financial exploitation have been estimated at around USD $2.9 billion [2]. Furthermore, many older adults face difficulties in managing finances, including suboptimal retirement decisions and vulnerability to financial scams [3]. These risks are particularly serious in later life, when opportunities to recover losses are limited and financial insecurity can negatively affect health and wellbeing [4].

The scale of this challenge is increasing. One reason is rapid population ageing, with projections indicating that by 2050, one in six people will be aged 65+ years [5]. At the same time, financial decision‐making has become more complex. For example, Australia's superannuation system (a compulsory, employment‐based retirement savings scheme) places substantial responsibility on individuals to manage retirement savings, requiring active engagement with financial products, investment choices, and drawdown strategies during a life stage when cognitive and physical resources may be declining [6]. Major financial decisions, including managing property, accessing retirement income, and planning for aged‐care costs, often converge in late midlife and early older adulthood and carry long‐term consequences for financial wellbeing, with potential for financial insecurity [7].

Navigating these demands requires adequate financial literacy, defined as the ability to process economic information and make informed decisions about financial planning, wealth accumulation, debt, and pensions [8]. Higher financial literacy is associated with greater retirement wealth, better investment decisions, and lower vulnerability to fraud [9]. Conversely, low financial literacy has been associated with costly borrowing behaviour, inadequate retirement planning, and reduced capacity to evaluate financial advice [9, 10]. Given the reliance on individual financial decision‐making in Australia and many other countries, understanding the distribution and determinants of financial literacy among older adults is especially important.

Cognitive function is a key determinant of financial literacy [11]. Ageing is associated with decline across multiple cognitive domains, including episodic memory, processing speed, executive function, and working memory, while other abilities such as crystallised knowledge tend to be more preserved [12, 13, 14]. Evidence suggests that cognitive performance is associated with financial literacy and financial capacity in older adults [15], and that cognitive decline predicts reductions in financial literacy over time [16]. However, most studies have examined a limited number of cognitive domains or relied on global cognitive screening measures [15, 16, 17], leaving uncertainty about which cognitive abilities are most implicated.

Further complexity arises from how financial literacy is conceptualised and measured. Studies have variously used subjective measures, in which individuals rate their own financial knowledge, and objective measures, in which knowledge is assessed through standardised tasks [18, 19, 20]. These approaches may capture distinct aspects of financial knowledge, but few studies have examined both subjective and objective measures within the same sample or compared their associations with cognitive performance measures. Similarly, while education and gender are established correlates of financial literacy, with evidence of systematic differences in financial knowledge, confidence, and engagement, it remains unclear whether they modify the relationship between cognitive performance and financial literacy [9, 21].

To address these gaps, we use data from Wave 5 of the PATH Through Life (PATH) Study, a population‐based Australian cohort of midlife adults. Previous analyses using PATH data showed that financial difficulties were associated with poorer cognitive performance [22, 23]. Here, we examine whether cognitive function is associated with financial literacy. The study aimed to: (1) characterise financial decision‐making experiences, including personal and substitute decision‐making; (2) describe the distribution of subjective and objective financial literacy; and (3) examine associations between cognitive functions and financial literacy, and whether these associations are modified by gender or education.

## 2 Methods

### 2.1 Study Design and Participants

The PATH Through Life (PATH) Study began in 1999 and enroled 7485 participants across three age cohorts: young adults (20–24 years), middle‐aged adults (40–46 years), and older adults (60–64 years) at baseline. Participants were randomly sampled from the electoral roll of the Australian Capital Territory and the nearby city of Queanbeyan, New South Wales. Cohort members have been followed up approximately every 4 years except for Wave 5, which occurred 7 years after Wave 4. Each wave comprised a broad assessment battery covering physical and mental health, cognitive function, lifestyle factors, and psychosocial wellbeing. Full details of the study design and cohort profile are reported elsewhere [24, 25].

The present study used cross‐sectional data from Wave 5 of the middle‐aged (i.e., 40–46 at baseline) cohort, collected between September 2019 and May 2020, at which point participants were 60–66 years old (mean 62.5) representing adults transitioning from late midlife to early older adulthood. Wave 5 was the first wave to introduce measures of financial decision‐making and financial literacy, making it the earliest timepoint at which our research questions can be examined. At Wave 5, participants were invited to complete two assessment components: (1) an online self‐complete questionnaire administered via Qualtrics, and (2) a 1.5‐h structured interview that included physical and cognitive assessments conducted in‐person. From March 2020, in‐person assessments were transitioned to telephone administration due to COVID‐19 public health restrictions (68% had completed their in‐person interview prior to this transition) [26]. Full details of the Wave 5 data collection procedures, including the impact of the COVID‐19 pandemic and 2019‐2020 bushfires on fieldwork, are reported elsewhere [26].

**FIGURE 1:**
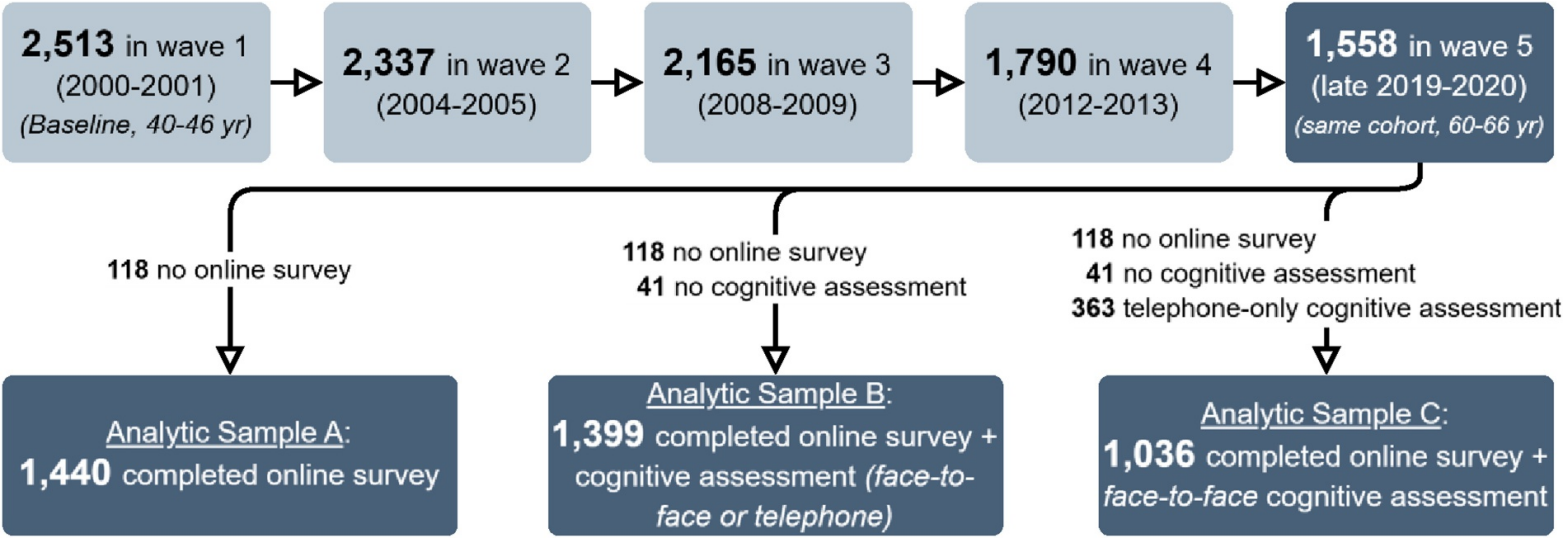
Flow diagram of study participants and composition of three analytic samples. All three analytic samples were derived from the same cohort of 1558 participants at wave 5. For cognitive assessments: (1) *Analytic sample A* was used to analyse the memory assessment clinics questionnaire. (2) *Analytic sample B* was used to analyse the California verbal learning test immediate recall, California verbal learning test delayed recall, symbol digit modalities test, trail making test part B, digit span backward, and spot‐the‐word test. (3) *Analytic sample C* was used to analyse the mini‐mental state examination, trail making test part A, and purdue pegboard test.

Of 1558 Wave 5 participants, three analytic samples were defined to maximise available sample size across cognitive assessments (Figure 1). Completion of the online self‐administered questionnaire was required for inclusion, as it provided measures of financial decision‐making and financial literacy, forming the base analytic sample (Sample A; *n* = 1440). From this sample, participants who completed any cognitive assessments were retained to form Sample B (*n* = 1399). Participants with only telephone‐based cognitive assessments were then excluded, resulting in Sample C (*n* = 1036).

### 2.2 Measures

#### 2.2.1 Financial Decision‐Making and Financial Literacy

Financial decision‐making experiences were retrospectively reported using a self‐administered online questionnaire, covering decisions made in the previous 5–6 years. The assessment included two areas: decisions made for oneself and decisions made on behalf of others. Participants reported whether they had faced significant financial decisions, their involvement, the involvement of others, and satisfaction with the process and outcome. For substitute decision‐making, participants indicated whether they had made decisions for someone else, the type of authority, and their experiences. Full survey items are outlined in Supporting Information S1: Table S1.

Financial literacy was assessed at Wave 5 using both subjective and objective indicators (Table 1), capturing participants' current knowledge.

1. *Subjective financial literacy* was measured with a single self‐rated item asking participants to rate their perceived financial knowledge on a 7‐point scale (1 = very low to 7 = very high).
2. *Objective financial literacy* was measured using four items adapted from Lusardi and Mitchell [27]. The first three items assessed core concepts (compound interest, inflation, and risk diversification), and a fourth item examined understanding of the relationship between interest rates and bond prices to tap more advanced knowledge [28]. Each item was scored dichotomously (1 = correct; 0 = incorrect), producing a sum score ranging from 0 to 4. Because a simple sum score assumes equal item difficulty, we derived a PRIDIT (Principal Component of Ridits)‐weighted score [29, 30]. PRIDIT applies a two‐step procedure: (1) ridit scoring, which transforms item responses based on the sample distribution and assigns greater weight to more informative items, and (2) principal component analysis to extract the first component as a composite literacy score. This approach provides a continuous, distribution‐referenced index with greater discriminability than the unweighted sum. PRIDIT computation was implemented using the *pridit* package in R.

**TABLE 1:** Survey items assessing subjective and objective financial literacy.

Subjective financial literacy	1	2	3	4	5	6	7
	Very low						Very high
On a scale from 1 to 7, how would you assess your overall financial knowledge?	☐	☐	☐	☐	☐	☐	☐

**Objective financial literacy**	**Response options**
Item 1: Compound interest Suppose you had $100 in a ‘no‐fees’ savings account and the interest rate was 2% per year. After 5 years, how much do you think you would have in the account if you left the money to grow?	☐ More than $102* ☐ Exactly $102 ☐ Less than $102 ☐ Do not know ☐ Refuse to answer
Item 2: Inflation Imagine that the interest rate on your savings account was 1% per year and inflation was 2% per year. After 1 year, how much would you be able to buy with the money in this account?	☐ More than today ☐ Exactly the same ☐ less than today* ☐ Do not know ☐ Refuse to answer
Item 3: Risk diversification Buying a single company's stock usually provides a safer return than a stock mutual fund.	☐ True ☐ False* ☐ Do not know ☐ Refuse to answer
Item 4: Bond price If interest rates rise, what will typically happen to bond prices?	☐ They will rise ☐ They will fall* ☐ They will stay the same ☐ There is no relationship between bond prices and the interest rate ☐ Do not know ☐ Refuse to answer

*Note:* Correct answers are indicated with an asterisk (*). “Do not know” and “Refuse to answer” responses were scored as incorrect.

Subjective and objective financial literacy were weakly correlated (*r* = 0.29), suggesting they are related but distinct.

#### 2.2.2 Cognitive Measures

The present study included all ten cognitive measures administered as part of the PATH Through Life Study cognitive battery at Wave 5. Measures were standardised to *z*‐scores (mean = 0, SD = 1) within each test using the Wave 5 sample distribution. Subjective memory impairment was assessed using the Memory Assessment Clinics Questionnaire (MAC‐Q), a six‐item self‐reported measure administered via the online questionnaire [31]. The remaining nine measures were interviewer‐administered cognitive tests assessing cognitive domains. Each test was analysed separately rather than combined into domain‐specific composites. These included the California Verbal Learning Test (CVLT) immediate and delayed recall (episodic memory) [32], Symbol Digit Modalities Test (SDMT) (processing speed and attention) [33], Trail Making Test Part A (TMT‐A) (processing speed and visual scanning) [34], Trail Making Test Part B (TMT‐B) (executive function and cognitive flexibility) [35], Digit Span Backwards (DSB) (working memory) [36], Mini‐Mental State Examination (MMSE) (global cognition) [37], Spot‐the‐Word Test (STW) (verbal knowledge/premorbid ability) [38], and Purdue Pegboard Test (PPT) (psychomotor speed and manual dexterity) [39].

As described in Section 2.1, data collection transitioned from face‐to‐face to telephone administration due to COVID‐19 restrictions [26]. Six tests could be administered by telephone (CVLT immediate and delayed recall, SDMT, TMT‐B, DSB, and STW), while three were conducted face‐to‐face only (MMSE, TMT‐A, and PPT).

#### 2.2.3 Covariates/Modifiers

Covariates included age, gender, race, years of education, weekly household income, and mode of cognitive test administration, all measured at Wave 5. Age was calculated from date of birth, and gender and race were self‐reported. Total years of education were derived from educational history items collected across waves and updated whenever participants reported additional qualifications. Weekly household income was reported in predefined categories. Mode of cognitive test was coded as face‐to‐face or telephone. Gender and years of education were additionally examined as effect modifiers, given prior evidence of systematic differences in financial literacy across demographic groups and their relevance to cognitive ageing and financial behaviour [9, 21].

### 2.3 Data Analysis

Statistical analyses were conducted using Stata v.19 (StataCorp, College Station, TX, USA) and R v.4.5.2 (R Foundation for Statistical Computing, Vienna, Austria). All reported *p*‐values were two‐sided, and the threshold for statistical significance was set at *α* = 0.05. As this study used secondary data from a large, population‐based cohort and included all available observations, sample size calculation was not performed.

#### 2.3.1 Missing Data

Missingness in the primary variables was generally low, with most variables ranging from 0.1% to 3%, with the exception of the Spot‐the‐Word test (8% missing) (Supporting Information S1: Table S2). To test whether data were missing completely at random (MCAR), we conducted Little's MCAR test and the assumption was not met (*p* < 0.001). Using only complete cases could therefore introduce selection bias. In addition, a complete‐case approach would have excluded up to ∼12% of participants for certain cognitive tests, reducing statistical power.

Therefore, missing data were imputed using the *missRanger* package in R, a machine learning approach that combines random forest imputation with predictive mean matching (PMM) [40, 41]. We specified 500 trees and 5 nearest neighbours for PMM. Briefly, missing values for each variable were iteratively predicted from all remaining variables using random forests, and plausible observed values were then matched and imputed. This method accommodates mixed variable types, complex non‐linear relationships without requiring parametric assumptions. Imputation was carried out for each analytic sample as defined in Section 2.1. All subsequent analyses used the imputed datasets.

#### 2.3.2 Primary Analysis

Participant characteristics were summarised using frequencies and percentages for categorical variables, and means with standard deviations (SD) or medians with interquartile ranges (IQR) for continuous variables, as appropriate. Associations between each cognitive measure (exposure) and financial literacy (outcome) were examined using linear regression models with MacKinnon‐White heteroskedasticity‐consistent standard errors (HC3) due to evidence of residual heteroskedasticity (Breusch‐Pagan test *p* < 0.05) [42]. Objective financial literacy was modelled using the PRIDIT‐weighted score in the primary analysis. All models were adjusted for the covariates listed in Section 2.2.3. Regression coefficients and 95% confidence intervals (CI) were reported. Because cognitive measures were *z*‐standardised, coefficients represent the expected change in financial literacy per 1‐SD difference in cognitive performance.

Effect modification by gender and years of education was examined by including interaction terms between each cognitive test and these variables. Interaction tests were conducted for all cognitive measures based on a priori hypotheses, regardless of whether the main associations were statistically significant. Interaction coefficients with 95% CI were reported. Interaction plots were presented to aid interpretation. Multiple testing was addressed using the Benjamini‐Hochberg false discovery rate procedure, and the corresponding adjusted *p*‐values were reported.

#### 2.3.3 Sensitivity Analysis

As a sensitivity analysis, an alternative objective financial literacy score was derived as the unweighted sum of correct answers. Regression models were re‐estimated using this measure to assess robustness of the findings.

## 3 Results

### 3.1 Participant Characteristics

The study included 1036‐1440 participants (Figure 1). The mean age was 62.5 years (SD 1.5), 53% were women, and 96% were Caucasian. Participants were highly educated (mean 15.2 years [SD 2.1]) and most reported moderate‐to‐high household incomes (Table 2).

**TABLE 2:** Summary of participant characteristics and financial literacy (*n* = 1440).

Characteristics	Value^a^
Age at wave 5, years	62.5 ± 1.5
Gender	
Men	673 (46.7)
Women	767 (53.3)
Race	
Caucasian	1390 (96.5)
All other groups^b^	50 (3.5)
Years of education	15.2 ± 2.1
Household income	
≤ $300/week	29 (2.0)
$300–$649/week	58 (4.0)
$650–$999/week	140 (9.7)
$1000–$1749/week	429 (29.8)
$1750–$2999/week	399 (27.7)
≥ $3000/week	265 (18.4)
Don't know	120 (8.3)
Subjective financial literacy	5.0 ± 1.2
Objective financial literacy	
Compound interest^c^	1329 (92.3)
Inflation^c^	1298 (90.1)
Risk diversification^c^	802 (55.7)
Bond price^c^	381 (26.5)
Sum of correct answers	2.6 ± 1.0
PRIDIT‐weighted score^d^	0.0 ± 0.03

^a^ Continuous variables are presented as Mean ± SD unless otherwise stated; categorical variables are presented as *n* (%).

^b^ All other groups included 29 Asian participants, 6 Aboriginal/Torres Strait Islander participants, and 15 participants from other backgrounds.

^c^ Number of participants who answered the item correctly.

^d^ PRIDIT‐weighted scores theoretically range from −1 to +1 (observed range in this study: −0.088 to 0.030); positive values indicate above‐average and negative values indicate below‐average financial literacy.

### 3.2 Financial Decision‐Making

**FIGURE 2:**
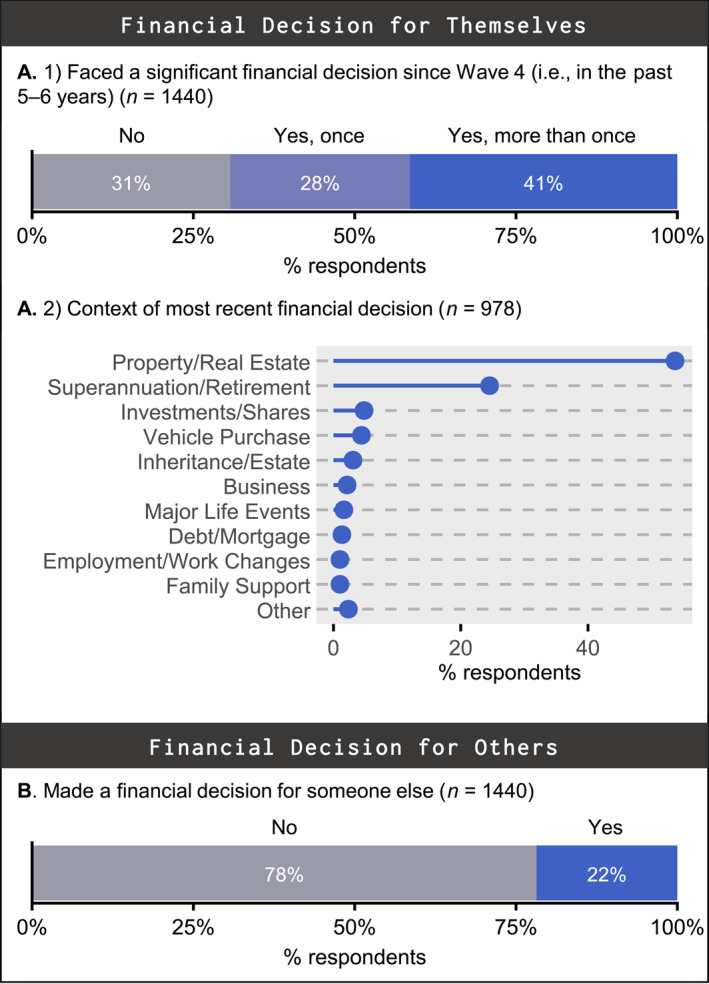
Descriptive characteristics of financial decision‐making. (A) (1) Whether participants had made a significant financial decision since wave 4 (assessed retrospectively over the previous 5–6 years) and the context of their most recent financial decision. (2) Free‐text responses describing the decision context were coded into categories. (B) Whether participants had made a financial decision on behalf of another person (i.e., acted as a substitute decision‐maker).

Over the preceding 5–6 years since the Wave 4 survey, 69% (*n* = 978) of participants reported facing at least one significant financial decision. Among these, the most common decisions involved property (54%) and superannuation or retirement planning (24%) (Figure 2A). When making financial decisions for themselves, participants most commonly involved their spouse (74%), but professional input was also frequently reported. Around 30% consulted a financial adviser, 14% an accountant, and 16% another professional. Notably, 8% reported involving no one else. Both participants' own involvement and the involvement of others generally fell within the ‘fairly involved’ to ‘very involved’ range on the Likert scale. Most participants also reported being satisfied with both the decision‐making process and the final outcome (Supporting Information S1: Figure S1A–D).

In the domain of financial decision‐making on behalf of others, 22% (*n* = 314) of participants reported having acted as a substitute decision‐maker (Figure 2B). The decision recipient was most commonly a parent (76%), and 67% of these substitute decisions were made under an ongoing legal authorisation. Many participants reported positive experiences in this role, including receiving clear information about their responsibilities. Most disagreed that acting as a substitute was stressful or that it exposed them to conflict with others (Supporting Information S1: Figure S1E–H).

### 3.3 Financial Literacy and Cognition

The mean self‐rated financial literacy score was 5.0 (SD 1.2) on a scale ranging from 1 (very low) to 7 (very high). For objective financial literacy, most participants correctly answered the questions on compound interest and inflation (over 90% correct). Correct responses were lower for risk diversification (56%) and lowest for the bond price question (27%). The mean number of correct answers across the four items was 2.6 (± 1.0) (Table 2). Among participants who reported making a financial decision (*n* = 978), levels of financial literacy were similar to those observed in the overall sample, with a mean self‐rated financial literacy of 5.2 (SD 1.1) and a mean of 2.7 (SD 0.9) correct answers on the objective financial literacy items (data not shown).

**FIGURE 3:**
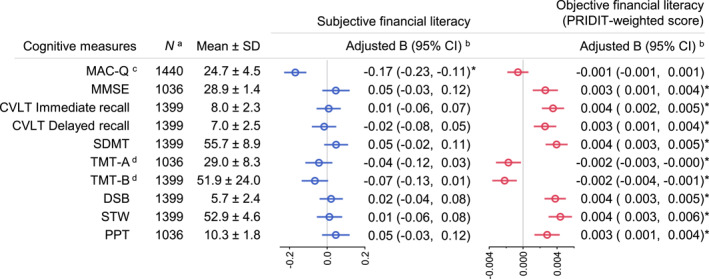
Association between cognition (exposure) and financial literacy (outcome). CVLT, california verbal learning test; DSB, digit span backward; MAC‐Q, memory assessment clinics questionnaire; MMSE, mini‐mental state examination; PPT, purdue pegboard test; SDMT, symbol digit modalities test; STW, spot‐the‐word test; TMT‐A, trail making test part A; TMT‐B, trail making test part B. An asterisk (*) indicates associations that remained statistically significant after adjustment for multiple testing using the Benjamini–Hochberg procedure (*p* < 0.05). ^a^ Sample sizes varied due to differences in assessment pathways (see Figure 1). ^b^ Adjusted for age, gender, race, years of education, household income, and mode of cognitive test administration. ^c^ Higher MAC‐Q value indicates more memory complaints (worse perceived memory). ^d^ Higher TMT‐A and TMT‐B values indicate longer completion time (worse performance).

After adjusting for covariates, higher MAC‐Q scores (indicating greater subjective memory impairment) were associated with lower subjective financial literacy (*B* = −0.17, 95% CI: −0.23, −0.11) (Figure 3). No associations between subjective financial literacy and other cognitive measures were observed. In contrast, objective financial literacy was associated with all cognitive measures assessed, but not with MAC‐Q. Specifically, better performance on measures of global cognition (MMSE), memory (CVLT immediate and delayed recall), processing speed (SDMT), executive function (TMT‐A and TMT‐B), working memory (DSB), verbal ability (STW), and manual dexterity (PPT) was independently associated with higher objective financial literacy scores (all *p* < 0.05 after Benjamini‐Hochberg correction; Figure 3). Sensitivity analysis using the number of correct responses produced results consistent with the main findings (Supporting Information S1: Figure S2).

**FIGURE 4:**
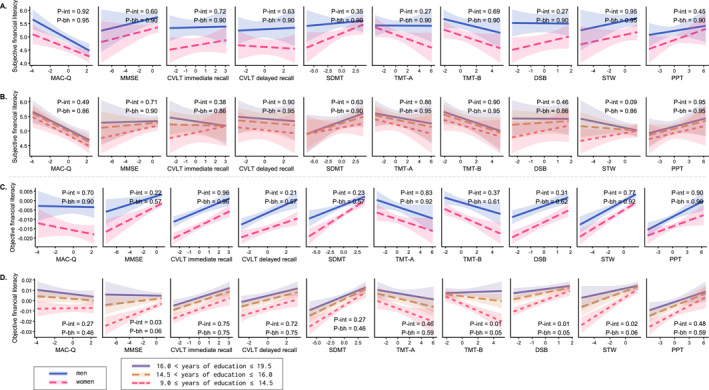
Interaction plots showing the associations between cognitive performance (exposure) and financial literacy (outcome) by gender and years of education. Panels A and B show the interaction between cognitive measures and subjective financial literacy by *gender* and *years of education*, respectively. Panels C and D show the interaction between cognitive measures and the PRIDIT‐weighted objective financial literacy score by *gender* and *years of education*, respectively. All models were adjusted for age, gender, race, years of education, household income, and mode of cognitive test administration. Years of education was modelled as a continuous variable. For ease of interpretation, the interaction plots display three representative categories: low (median of the lowest tertile = 13 years), medium (median of the middle tertile = 16 years), and high education (median of the highest tertile = 18 years). **P‐int** indicates the *p*‐value for the interaction term, and **P‐bh** indicates the *p*‐value after controlling for multiple testing using the Benjamini‐Hochberg false discovery rate procedure. Detailed results, including interaction coefficients and 95% confidence intervals, are reported in Supporting Information S1: Table S3.

We then examined whether gender or education modified the associations between cognitive measures and financial literacy. For subjective financial literacy, neither gender nor education showed significant interaction (Figure 4A–B; Supporting Information S1: Table S3). For objective financial literacy, no significant interactions with gender were observed. However, education modified the relationship between objective financial literacy and cognition, with significant interaction terms for MMSE, TMT‐B, DSB, and STW (TMT‐B and DSB remained significant after Benjamini‐Hochberg correction) (Figure 4C–D; Supporting Information S1: Table S3).

## 4 Discussion

### 4.1 Main Findings

In this community‐based sample of Australian adults transitioning from late midlife to early older adulthood, financial decision‐making was common. Approximately 7 in 10 participants reported making at least one major financial decision in the recent past (often related to property or retirement savings). Around 1 in 5 had also acted as a substitute financial decision‐maker, typically for a parent. Despite this high prevalence, financial literacy varied. While self‐rated literacy was generally high, objective literacy showed limitations in more complex financial concepts (such as risk diversification and bond pricing). Subjective financial literacy was associated only with self‐reported memory impairment. In contrast, objective financial literacy was associated with multiple cognitive domains, including global cognition, memory, processing speed, executive function, working memory, verbal ability, and manual dexterity. Education modified some of these relationships, particularly for executive function and working memory, with evidence of attenuation in the association between cognitive function and objective financial literacy among those with higher education. No interaction with gender was found.

### 4.2 Interpretation

Financial decision‐making was highly prevalent in this cohort, consistent with financial responsibilities that arise during this life stage. Individuals often shift from wealth accumulation to retirement planning and asset management, including superannuation access and property transitions such as downsizing [43]. Within Australia's retirement income system in which individuals bear responsibility for managing superannuation savings, these decisions are both expected and consequential [44].

The finding that 1 in 5 participants had acted as a substitute financial decision‐maker highlights the intergenerational financial responsibilities experienced at this life stage. Many individuals may be managing their own financial transitions while simultaneously supporting older family members. Although substitute decision‐making is recognised, its prevalence in community‐based Australian samples in late midlife has rarely been quantified. These results therefore provide descriptive evidence of the broader social context in which financial decisions are made.

Another finding was the discrepancy between subjective and objective financial literacy. Participants generally rated their financial knowledge highly, but objective assessments showed limited understanding. This mismatch suggests an overconfidence in financial self‐assessment, consistent with international evidence. Data from Organisation for Economic Co‐operation and Development (OECD) countries indicate that many adults overestimate their financial knowledge relative to their objective performance [9], with similar findings reported in Australia [7]. A study among older adults has shown that overconfidence in financial knowledge was associated with greater tolerance for financial risk, although whether this translates into poorer real‐world financial decisions is unclear [45]. Financial literacy may therefore be best understood as a multidimensional construct including both perceived and actual abilities. Future research should examine whether targeted decision support, including timely and relevant information or trusted guidance during complex financial decisions, can support decision‐making among individuals with lower cognitive function or educational attainment.

Subjective financial literacy was linked only to self‐reported memory impairment (assessed by MAC‐Q), suggesting it may represent metacognitive self‐appraisal rather than actual knowledge. The MAC‐Q captures perceived memory functioning [31], and a similar process may underlie subjective financial literacy. Therefore, individuals who perceive memory difficulties may rate their financial knowledge lower, even in the absence of knowledge deficits. This interpretation is supported by prior work showing that subjective memory complaints are often weakly related to objective memory performance [46]. Indeed, MAC‐Q scores showed negligible correlation with objective memory measures in the present dataset (immediate recall *r* = −0.04; delayed recall *r* = −0.05).

In contrast, objective financial literacy had associations with multiple cognitive measures. While previous studies have primarily focused on global cognition, our findings extend this literature by examining associations across specific cognitive domains [15, 16, 17]. Although cognitive measures have overlapping task demands, domain‐specific analyses provide a more detailed characterisation of the cognitive abilities associated with financial literacy.

Episodic memory, assessed using CVLT immediate and delayed recall, was associated with financial literacy, consistent with prior evidence linking memory performance to financial literacy in older adults without dementia [15]. Evidence from the same cohort further indicated that, among individuals with mild cognitive impairment, financial literacy remained associated with memory performance [17]. Episodic memory supports the encoding and retrieval of financial information, including previously learnt concepts and past decisions [47]. Impairments in these processes may limit the ability to retain and apply financial knowledge, thereby reducing objective financial literacy.

Processing speed was also related to financial literacy across both SDMT and TMT‐A measures. Slower processing may reduce the efficiency with which individuals integrate and evaluate financial information, particularly when tasks involve multiple steps or time constraints. A prior study from the US has similarly linked perceptual speed to financial literacy in older adults [48]. Our findings extend this evidence to an Australian community‐based sample, supporting the generalisability of this relationship.

Executive function, measured using TMT‐B, was also associated with financial literacy. This domain supports planning, cognitive flexibility, and rule‐based reasoning, all of which are necessary for evaluating financial options and making complex decisions. Longitudinal evidence suggests that decline in executive function reduced financial literacy over time, independent of other cognitive domains [15]. Similar findings have been reported in Greek clinical populations, where poorer executive function was associated with reduced financial capacity among older adults with mild cognitive impairment and dementia, although these studies examined financial capacity rather than financial literacy [49, 50].

Working memory supports the ability to hold and manipulate information during multi‐step financial reasoning. Its role may be particularly important for complex financial concepts, such as risk diversification and bond pricing, which require active information processing rather than reliance on stored knowledge. Consistent with this, a study in cognitively unimpaired older adults showed that financial capacity was associated with working‐memory updating alongside broader cognitive abilities [51].

The association between STW scores and financial literacy was expected. The STW measures crystallised cognition (accumulated verbal knowledge built through lifetime education and experience) and is validated as a stable estimate of premorbid ability [52]. Prior evidence indicated that vocabulary and semantic memory were associated with financial literacy in older adults [21], and that preserved crystallised intelligence may buffer age‐related declines in financial literacy [53]. Given the relatively narrow age range of this sample (60–66 years), in which crystallised abilities are typically maintained [54], the observed association aligns with theoretical expectations. The association persisting after adjusting for education underlines that the depth of semantic knowledge, above and beyond years of formal education, contributes to financial literacy.

The association between PPT performance and objective financial literacy warrants careful interpretation. The PPT measures fine motor speed and bimanual coordination [55], abilities that are unlikely to directly influence financial reasoning. Instead, this relationship may reflect broader underlying brain health. Differences in white matter structure, cerebrovascular health, and neural connectivity can influence both motor performance and higher‐order cognitive performance [55, 56, 57]. Although the analyses adjusted for chronological age, individuals of the same age can differ substantially in underlying brain health [58]. This within‐age variation may contribute to the observed association. Prior research showed that motor and cognitive functions are known to decline in parallel in older adults as part of a broader neurobiological ageing process [59, 60]. Within this context, PPT performance may act as a marker of overall brain health rather than a direct contributor to financial reasoning.

Education and literacy have been identified as key determinants of financial literacy and financial capacity across diverse populations [61]. In this study, education modified cognition‐financial literacy relationship, with stronger associations observed among individuals with lower educational attainment, particularly for executive function and working memory. This aligns with the cognitive reserve concept [62], whereby higher education could buffer the consequences of reduced cognitive performance. In the context of financial literacy, individuals with higher education may draw on accumulated knowledge and established decision strategies to compensate for reduced executive or working memory ability. However, evidence for this interaction effect remains mixed. While some studies report significant interactions between education and cognitive performance in relation to financial literacy [17], others have not observed such effects [21]. Education may still play a protective role in maintaining financial literacy, although the extent of this effect may vary across populations and study designs.

No evidence of gender‐based effect modification was observed. While women often report lower financial literacy overall [63, 64], the relationship between cognitive performance and financial literacy appeared consistent across genders. In other words, cognitive function appears to influence financial literacy similarly in men and women, despite differences in baseline literacy levels. Therefore, the cognitive processes underpinning financial literacy may operate similarly in men and women, even where baseline literacy differs.

### 4.3 Implications

Our findings highlight the importance of considering cognitive function in financial policy and public health strategies for adults in mid‐to‐late life. Individuals with lower education appear more vulnerable to the effects of reduced cognitive function on financial literacy, indicating a need for targeted support. Brief screening of financial literacy, alongside practical education on complex financial concepts such as retirement income products and risk diversification, may be useful. These strategies may also help reduce vulnerability to financial scams and exploitation.

At the same time, financial literacy is likely shaped across the life course. Educational attainment likely reflects broader socioeconomic conditions and cognitive reserve accumulated earlier in life. This means that short‐term education alone in later life is unlikely to fully offset these differences. More effective approaches may involve system‐level changes, including clearer communication, simplified decision pathways, and built‐in safeguards within superannuation systems.

To clarify, strengthening financial education remains important before individuals reach the stage where major financial decisions are made, such as retirement planning and asset allocation. Complementing this, improving accessibility and ongoing support in later life, when both personal and substitute financial decision‐making are common, may improve decision‐making quality and help maintain financial independence.

### 4.4 Strengths and Limitations

Our study has many strengths. The use of the PATH Through Life cohort provides a well‐characterised, population‐based community sample with high‐quality data and standardised cognitive assessments [24, 25, 26]. The distinction between subjective and objective financial literacy, and their contrasting cognitive performance correlates, contributes to a clearer understanding of the nature of self‐rated knowledge versus actual financial literacy. The assessment of both personal and substitute financial decision‐making in late midlife adults transitioning into early older adulthood addresses an important gap in the literature.

Several limitations should also be acknowledged. *First*, the cross‐sectional design precludes causal inference. Although cognitive performance may influence financial literacy, financially literate individuals may also engage in cognitively stimulating financial activities that contribute to cognitive maintenance. In addition, financial decision‐making experiences were retrospectively reported for the preceding 5–6 years, whereas financial literacy captured their current knowledge assessed at Wave 5. This temporal mismatch limits the analysis and interpretation of any association between financial literacy and financial decision‐making, as it cannot be determined whether financial literacy preceded or followed these experiences. Longitudinal follow‐up will be necessary to establish temporal directionality. *Second*, the cohort's relatively high educational attainment and socioeconomic position may limit generalisability and could attenuate observed associations due to ceiling effects in financial literacy. In addition, the use of within‐cohort standardisation for cognitive scores means that *z*‐scores capture relative performance within this higher‐functioning sample, which may further limit comparability with general population norms. *Third*, the financial literacy measure assessed knowledge of financial concepts rather than real‐world financial behaviour. In addition, as the measure was based on a multiple‐choice format, performance may partly reflect test‐taking strategies associated with higher educational attainment rather than financial knowledge alone. Future work linking survey data with administrative or financial records may help address this limitation. *Fourth*, although gender was tested as an effect modifier, the study could not assess factors such as lifetime financial socialisation or partner specialisation in financial management, which have been suggested to contribute to gender differences in financial literacy [65]. *Finally*, attrition bias may lead to under‐representation of individuals with lower cognitive function or financial literacy, meaning that associations reported here may be conservative.

## 5 Conclusions

Financial decision‐making was common among adults transitioning from late midlife to early older adulthood. Objective financial literacy was associated with cognitive performance across multiple domains, whereas subjective financial literacy was associated only with subjective memory complaints. Subjective financial literacy did not correspond to objective financial knowledge. Education modified some of these associations, with weaker cognition–objective financial literacy relationships observed among individuals with higher educational attainment. Overall, the key message is that cognitive function influences financial literacy, with implications for managing complex financial decisions. These findings highlight the importance of considering both cognitive performance and objective financial literacy when evaluating financial capability.

## Author Contributions

**Htet Lin Htun:** conceptualization, methodology, formal analysis, investigation, writing – original draft, writing – review and editing. **Craig Sinclair:** investigation, resources, writing – review and editing. **Ranmalee Eramudugolla:** investigation, resources, writing – review and editing. **Kaarin J. Anstey:** conceptualization, supervision, funding acquisition, writing – review and editing.

## Funding

The authors acknowledge financial support from the Australian Research Council Centre of Excellence in Population Ageing Research (CEPAR) project number CE170100005.

## Ethics Statement

The Wave 5 follow‐up of the PATH 40s cohort was approved by the UNSW Human Research Ethics Committee (HREC; Protocols: HC180505 and HC180518). Written informed consent was obtained from all participants.

## Conflicts of Interest

The authors declare no conflicts of interest.

## Supporting information


Supporting Information S1

## Data Availability

The data supporting the findings of this study are available from the corresponding author upon reasonable request. The PATH study is co‐hosted by UNSW Sydney and the Australian National University, and data access is subject to study governance procedures (https://www.pathstudy.org.au/; info@pathstudy.org.au).
