# Supplementary material for: Financial Decision‐Making, Financial Literacy, and Cognitive Function: Evidence From the PATH Through Life Study
Source: Int J Geriatr Psychiatry. 2026 Aug 17;41(8):e70248. doi: 10.1002/gps.70248 (PMC13479054; doi:10.1002/gps.70248)
Supplement: Supplementary file 1 [file GPS-41-e70248-s001.pdf]

Financial Decision-Making, Financial Literacy, and Cognitive Function: Evidence from the PATH Through Life Study

Table of Contents

Table S1. Survey items assessing financial decision characteristics

Table S2. Distribution of missing data in study outcome and main exposure variables

Table S3. Interaction effects of financial literacy with gender and years of education on cognitive measures

Figure S1. Financial decision-making characteristics across two domains

Figure S2. Association between cognitive performance measures and objective financial literacy measured by number of correct responses

Table S1. Survey items assessing financial decision characteristics

Item	Question	Response Options
**Section A: Financial Decision for Themselves**		
A1	In the time since your most recent PATH survey, have you been faced with a significant financial decision (for example, the purchase or sale of a house, or other valuable asset, or a major decision about your savings or investments)?	No<br>Yes, once<br>Yes, more than once
A2	Briefly describe what the most recent of these significant financial decisions was.	Open text
A3	Overall, how involved did you feel that you were in the decision?	Not at all involved<br>A little involved<br>Fairly involved<br>Very involved
A4	Think about the other people who were involved in the decision. (Tick all that apply)	Spouse or partner<br>Child/children<br>Other family members<br>Friend/s<br>Financial Adviser<br>Accountant<br>Other professional<br>Other (specify)<br>None
A5	Thinking about just this person's involvement in the decision-making, how involved were they in:<br>1) Identifying that a decision needed to be made<br>2) Providing information or helping in the search for information<br>3) Helping you understand your options<br>4) Helping you to choose or decide<br>5) Helping to put the decision into practice	Not at all involved<br>A little involved<br>Fairly involved<br>Very involved
A6	Overall, how satisfied were you with the way the decision was made?	Very satisfied<br>Somewhat satisfied<br>Neither<br>Somewhat dissatisfied<br>Very dissatisfied
A7	Overall, how satisfied were you with how the decision turned out?	Very satisfied<br>Somewhat satisfied<br>Neither<br>Somewhat dissatisfied<br>Very dissatisfied
**Section B: Financial Decision for Others**		
B1	Have you been required to make financial decisions for someone else due to their inability?	No<br>Yes
B2	Who did you make financial decisions for? (Select all that apply)	Parent<br>Spouse/partner<br>Child<br>Sibling<br>Other family member<br>Friend<br>Other (specify)
B3	In what capacity were you making financial decisions for someone else?<br>○ Under a temporary legal authorisation (e.g., General Power of Attorney)<br>○ Under an ongoing legal authorisation (e.g., Enduring Power of Attorney)<br>○ Under an appointed authorisation (e.g., Financial Administration Order from a court or tribunal)<br>○ Existing arrangement (e.g., as a joint signatory on a bank account with spouse/partner)<br>○ Informal arrangement (e.g., informal family agreement)	

	○ The person asked me to make the decision or transaction for them (e.g., by sharing their bank account personal identification number)		
	○ Don't know		
B4	Please rate your agreement with the following statements regarding decisions made for others.		
	1) I have received clear information about my role and responsibilities as a financial decision-maker		Strongly disagree<br>Disagree
	2) I found it stressful to make decisions for someone else		Unsure
	3) Being the decision-maker for someone else exposed me to arguments and/or conflict with others		Agree<br>Strongly agree

Table S2. Distribution of missing data in study outcome and main exposure variables

Variables	No. missing/ No. in cohort	% missing
**Subjective Financial Literacy**	27/1440	1.9
**Objective Financial Literacy**		
Item 1: Interest rate	27/1440	1.9
Item 2: Inflation	27/1440	1.9
Item 3: Risk diversification	29/1440	2.0
Item 4: Bond price	27/1440	1.9
**Financial Decision**		
Faced a significant financial decision	28/1440	1.9
Made a financial decision for someone else	28/1440	1.9
**Subjective Memory**		
Memory Assessment Clinics Questionnaire	45/1440	3.1
**Cognitive Tests (face-to-face or telephone administered)**		
CVLT Immediate recall	1/1399	0.1
CVLT Delayed recall	2/1399	0.1
Symbol Digit Modalities Test	11/1399	0.8
Digit Span Backward	2/1399	0.1
Spot-the-Word	113/1399	8.1
Trail Making Test B	3/1399	0.2
**Cognitive Tests (face-to-face administered only)**		
Trail Making Test A	0/1036	0.0
Mini-Mental State Examination	0/1036	0.0
Purdue Pegboard Test	6/1036	0.6

Note. Missing data are reported within each analytic cohort; therefore, denominators vary across measures.

Table S3. Interaction effects of financial literacy with gender and years of education on cognitive measures

Cognitive Measure	B coef.	LCI	UCI	P-int	P-BH
**Subjective Financial Literacy x Gender**					
MAC-Q	0.0319	-0.0859	0.1497	0.60	0.90
MMSE	0.0090	-0.1611	0.1791	0.92	0.95
CVLT Immediate recall	0.0235	-0.1031	0.1502	0.72	0.90
CVLT Delayed recall	-0.0310	-0.1552	0.0933	0.62	0.90
SDMT	0.0570	-0.0636	0.1775	0.35	0.90
TMT-A	-0.0810	-0.2234	0.0615	0.26	0.90
TMT-B	-0.0245	-0.1463	0.0973	0.69	0.90
DSB	0.0651	-0.0507	0.1808	0.27	0.90
STW	-0.0039	-0.1286	0.1208	0.95	0.95
PPT	0.0548	-0.0872	0.1968	0.45	0.90
**Subjective Financial Literacy x Years of education**					
MAC-Q	-0.0053	-0.0329	0.0223	0.71	0.90
MMSE	-0.0127	-0.0488	0.0233	0.49	0.86
CVLT Immediate recall	-0.0130	-0.0424	0.0163	0.38	0.86
CVLT Delayed recall	0.0019	-0.0268	0.0306	0.90	0.95
SDMT	0.0068	-0.0208	0.0343	0.63	0.90
TMT-A	0.0034	-0.0338	0.0406	0.86	0.95
TMT-B	-0.0019	-0.0310	0.0272	0.90	0.95
DSB	-0.0103	-0.0380	0.0174	0.46	0.86
STW	-0.0241	-0.0522	0.0041	0.09	0.86
PPT	0.0012	-0.0326	0.0349	0.95	0.95
**Objective Financial Literacy x Gender**					
MAC-Q	-0.0005	-0.0030	0.0020	0.70	0.90
MMSE	0.0020	-0.0012	0.0053	0.22	0.57
CVLT Immediate recall	0.0001	-0.0025	0.0026	0.96	0.96
CVLT Delayed recall	-0.0016	-0.0041	0.0009	0.21	0.57
SDMT	0.0015	-0.0009	0.0040	0.23	0.57
TMT-A	0.0003	-0.0026	0.0032	0.83	0.92
TMT-B	-0.0013	-0.0041	0.0015	0.37	0.61
DSB	0.0013	-0.0012	0.0038	0.31	0.62
STW	0.0004	-0.0025	0.0034	0.77	0.92
PPT	-0.0002	-0.0034	0.0030	0.90	0.96
**Objective Financial Literacy x Years of education**					
MAC-Q	-0.0004	-0.0010	0.0003	0.27	0.46
MMSE	-0.0009	-0.0018	-0.0001	**0.03**	0.06
CVLT Immediate recall	-0.0001	-0.0007	0.0005	0.75	0.75
CVLT Delayed recall	-0.0001	-0.0007	0.0005	0.72	0.75
SDMT	-0.0004	-0.0010	0.0003	0.27	0.46
TMT-A	0.0003	-0.0005	0.0010	0.46	0.60
TMT-B	0.0010	0.0003	0.0016	**0.01**	**0.05**
DSB	-0.0008	-0.0014	-0.0002	**0.01**	**0.05**
STW	-0.0008	-0.0015	-0.0001	**0.02**	0.06
PPT	-0.0003	-0.0011	0.0005	0.48	0.60

Interaction coefficients (B) with 95% confidence intervals (LCI, UCI) from linear regression models examining whether associations between cognitive measures and financial literacy are modified by gender and years of education. All models were adjusted for age, gender, race, years of education, household income, and mode of cognitive test administration. Robust standard errors were estimated using the HC3 method.
P-int indicates the p-value for the interaction term. P-BH indicates p-values adjusted for multiple testing using the Benjamini–Hochberg false discovery rate procedure.
Abbreviations: CVLT, California Verbal Learning Test; DSB, Digit Span Backward; MAC-Q, Memory Assessment Clinics Questionnaire; MMSE, Mini-Mental State Examination; PPT, Purdue Pegboard Test; SDMT, Symbol Digit Modalities Test; STW, Spot-the-Word test; TMT-A, Trail Making Test Part A; TMT-B, Trail Making Test Part B.

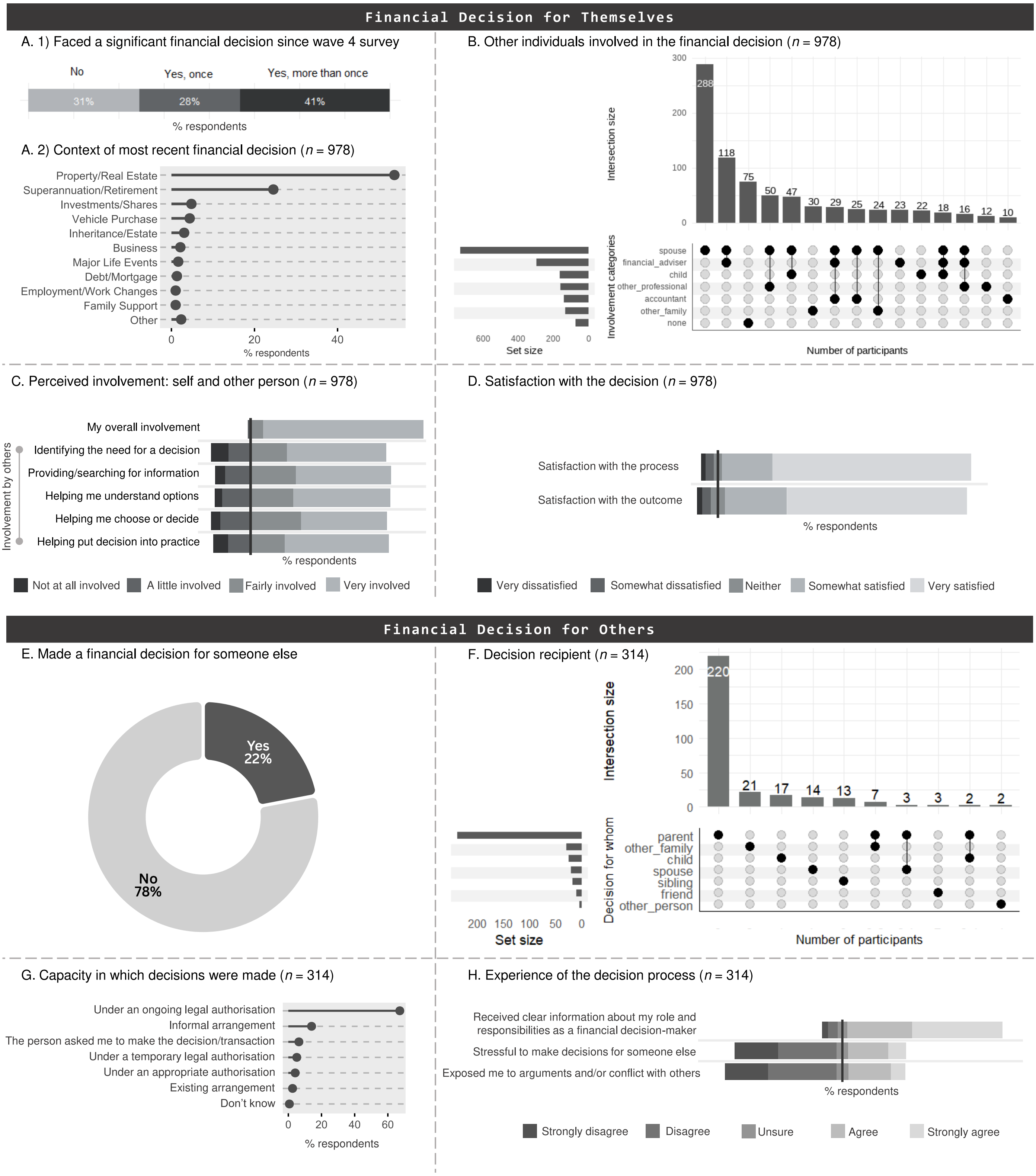

Figure S1. Financial decision-making characteristics across two domains

Panels A–D present characteristics related to financial decisions for themselves.
Panels E–H present characteristics related to financial decisions for others.

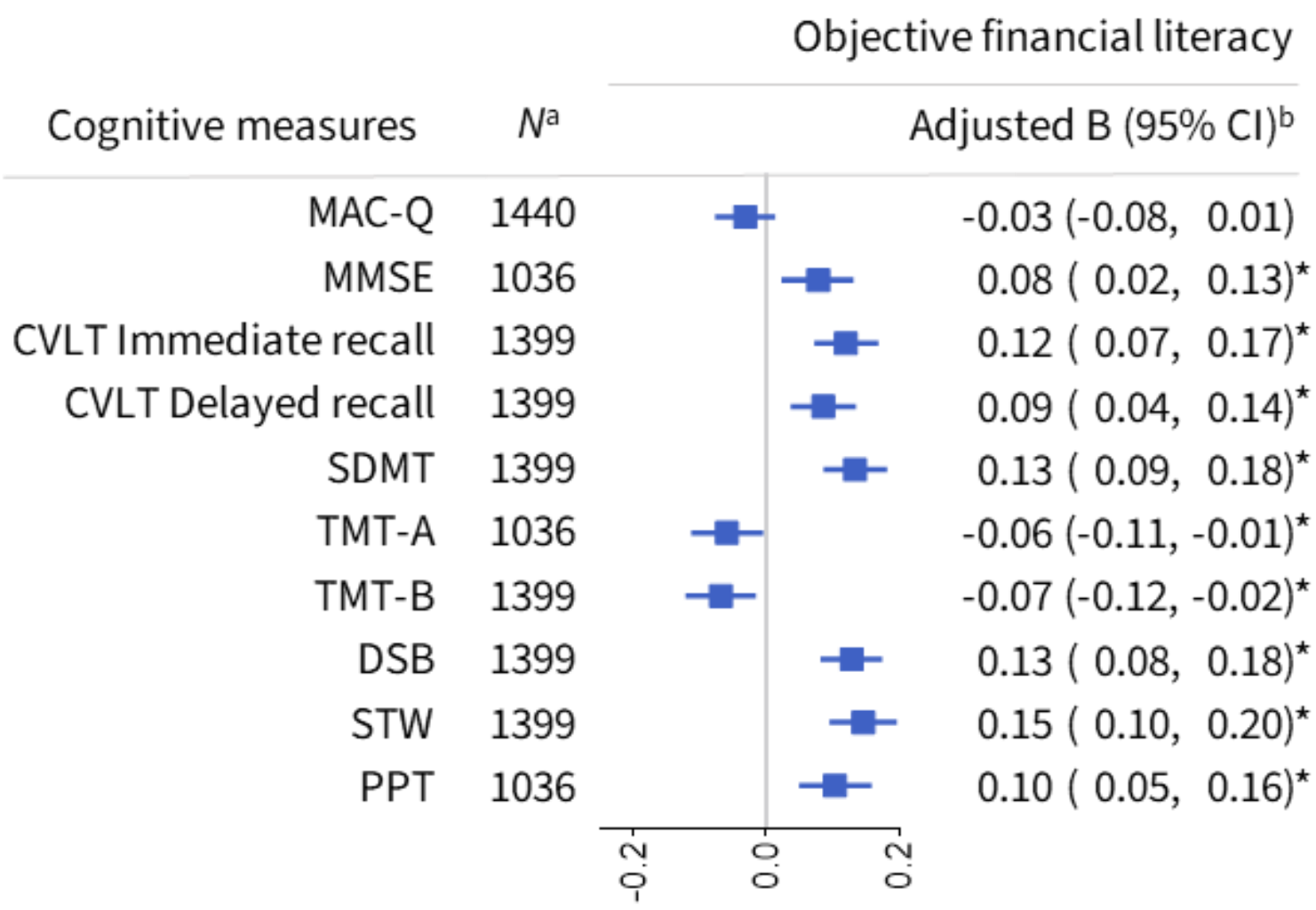

Figure S2. Association between cognitive performance measures and objective financial literacy measured by number of correct responses

Abbreviations: CVLT, California Verbal Learning Test; DSB, Digit Span Backward; MAC-Q, Memory Assessment Clinics Questionnaire; MMSE, Mini-Mental State Examination; PPT, Purdue Pegboard Test; SDMT, Symbol Digit Modalities Test; STW, Spot-the-Word test; TMT-A, Trail Making Test Part A; TMT-B, Trail Making Test Part B.

An asterisk (*) indicates estimates that remained statistically significant after adjustment for multiple testing using the Benjamini–Hochberg procedure ($P < 0.05$).

[a] Sample sizes vary due to differences in assessment pathways (see Figure 1).

[b] Adjusted for age, gender, race, years of education, household income and mode of cognitive test administration.
